# Associations between mobile phone involvement, BMI levels, and sleep quality among Chinese university students: evidence from a multi-regional large-scale survey

**DOI:** 10.3389/fpubh.2025.1533613

**Published:** 2025-02-17

**Authors:** Yukun Lu, Haodong Tian, Wentao Shi, Haowei Liu, Jinlong Wu, Yunfei Tao, Li Peng

**Affiliations:** ^1^College of Physical Education, Southwest University, Chongqing, China; ^2^College of Physical Education, Xinjiang Hetian College, Hetian, China; ^3^Key Laboratory of Physical Fitness Evaluation and Sports Function Monitoring of General Administration of Sport of China, Chongqing, China

**Keywords:** Chinese university students, mobile phone involvement, BMI levels, sleep quality, relationships

## Abstract

**Objective:**

This study aims to explore the association between mobile phone involvement, body mass index (BMI) levels, and the sleep quality of Chinese university students.

**Methods:**

Using a cluster sampling method, we selected 17,085 university students from three universities in eastern, central, and western China as the study subjects. Demographic information such as age and sex were collected. The Pittsburgh Sleep Quality Index (PSQI) and the Mobile Phone Involvement Questionnaire (MPIQ) were utilized to measure their sleep quality scores and mobile phone involvement scores, respectively. Pearson correlation analysis, two-way ANOVA, and multiple linear regression were employed to examine the relationship between BMI levels, mobile phone involvement, and sleep quality.

**Results:**

The results show that 15.87% (2,712 participants) are classified as overweight, and 18.45% (3,151 participants) are classified as obese. Additionally, 35.87% (6,125 participants) exhibit mobile phone involvement, while 57.94% (9,899 participants) reported poor sleep quality. Pearson correlation analysis indicates a significant negative correlation (*p* < 0.01) between sleep quality and both BMI levels and mobile phone involvement. Two-way ANOVA shows the significant effect of BMI levels (*p* < 0.001) and mobile phone involvement (*p* < 0.001) on sleep quality, and there is no interaction effect between the two. Additionally, the sleep quality of overweight and obese individuals is significantly poorer than that of those with normal weight (*p* < 0.05), while the sleep quality of overweight individuals is significantly lower than that of obese individuals (*p* < 0.05). Multiple linear regression analysis indicates that, after controlling for age and gender, both BMI (*β* = −2.69) levels and mobile phone involvement (*β* = −1.34) are significantly negatively associated with sleep quality (*p* < 0.001), accounting for 19% of the variance in poor sleep quality.

**Conclusion:**

This study found that BMI levels and mobile phone involvement are both independently associated with sleep quality among Chinese university students. However, among individuals with excess BMI, although their sleep quality is worse than individuals with normal weight, overweight individuals may have poorer sleep quality than obese individuals. This study also revealed high rates of overweight and obesity, with over half of participants reporting poor sleep quality, highlighting the need for targeted interventions to address weight management and mobile phone usage to improve sleep health in this population.

## 1 Introduction

University students are at a crucial stage of physiological and psychological development. Cultivating proper sleep habits during this period contributes to their mental health (1), and physical health (2), and academic performance (3). However, irregular sleep patterns are making university students a vulnerable group with poor sleep quality (4), and the resulting chronic sleep deprivation can lead to a range of negative impact on their overall health. Especially in China, the prevalence of poor sleep quality among college students is notable. Numerous studies have indicated a strong correlation between low sleep quality and psychological health issues, including stress, anxiety, and depression, which may further reduce students’ social relationships and health lifestyle (5–8). Furthermore, sleep quality is associated with the occurrence of diseases such as impaired cardiovascular function (9), abnormal body composition (10), and gastrointestinal dysfunction (11). As a result, improving sleep quality among university students has become a critical area of research for educational and health-related authorities.

In 2014, Word health Organization (WHO) emphasized the impact of information and communication technology use on public health and excessive behaviors (12). And problematic mobile phone use (PMPU), which is generally conceptualized as a behavioral addiction, has raised worldwide concerns for being considered as a potential health issue. However, based on the three key characteristics of addictive behavior (control, tolerance, and withdrawal) proposed in the DSM-5, there is currently insufficient evidence to support PMPU as a behavioral addiction. Consequently, Billieux et al. (13) proposed a theoretical framework which highlights that PMPU is a heterogeneous and multifaceted pathological condition, and the use of the concept of “mobile phone addiction (MPA)” should still be approached with caution. In contrast, mobile phone involvement, which is defined as excessive use when daily usage exceeds 4 hours (14, 15), may be more suitable. Up to 2020, the mobile phone users are estimated to be 1.05 billion, with young people aged 20–29 accounting for 17.2% (16). This indicated an increasingly severe phenomenon of mobile phone involvement among university students (17), which not only affects students’ academic performance and social interactions but also poses sleep-related problems (18, 19). For instance, nighttime phone use can delay sleep onset, shorten sleep duration, and reduce sleep quality (20, 21), which may involve a range of physiological and cognitive mechanisms. Specifically, the strong impact of blue light exposure from mobile phone on circadian rhythms (22), the cognitive arousal caused by using phones (23), and potential impairments in executive function (24) are significant factors contributing to these issues. Additionally, chronic sleep deprivation or poor sleep quality can impair emotional regulation and increase the risk of negative emotions such as depression and anxiety (25). Individuals may resort to using their phones to alleviate these negative emotions, seeking social support and self-comfort. Frequent use of phones for emotional regulation can, in turn, lead to involvement and addiction (26).

BMI is a typical indicator for assessing obesity, which is crucial for overall health status (27). To date, studies have shown a series of biological impacts of obesity on sleep duration (28), and sleep-related issues such as Obstructive Sleep Apnea (OSA) (29, 30), gastroesophageal reflux during nighttime (31), impaired melatonin synthesis (32), and nervous system imbalance (33) have been proved to be closely associated with fat accumulation. Consequently, individuals with higher BMI generally experiencing poorer sleep (34, 35). Besides sleep quality, a latest cross-sectional clinical interview has reported the close relationship between BMI and mobile phone involvement (36). Furthermore, obesity and mobile phone dependency may cooperatively induce damage in brain structures related to cognitive control and emotional regulation (37).

Current studies have separately examined the impact of BMI and mobile phone involvement on the sleep quality of university students (38–40). However, the interactive effects of BMI and mobile phone involvement on sleep quality, along with their relationship among university students, still requires further validation. Therefore, this study aims to respond these problems by including a large sample of participants and observing the sleep quality with varying levels of mobile phone involvement (non-involved, involved) and BMI (ordinary, overweight, obese). These findings can help offer theoretical insights in designing effective interventions on sleep-related issues of university students.

## 2 Materials and methods

This study was conducted from August 4, 2023, to June 3, 2024 among students from three universities located in Eastern, Central, and Western China. A cluster sampling method was employed to ensure a representative sample from diverse geographical regions. Specifically, the study was structured around three defined clusters based on these regions. From each region, one university was randomly selected to represent its respective area. The selection process utilized a random number table, guaranteeing that each university had an equal opportunity to be included in the study. Ethical approval for the study was obtained from the Southwest University hospital (SWU-ETH-2023-07-17-005).

### 2.1 Participants

At each university, the physical fitness testing phases across two semesters were primarily selected for the concentrated recruitment of participants. In addition, we supplemented the sample before and after the physical education classes in the selected universities. Inclusion criteria were: (1) voluntary participation, (2) undergraduate Chinese students (aged 22 or younger) (3) normal communication and comprehension abilities. Exclusion criteria were: (1) a history of diagnosed sleep disorders, (2) the Use of sleeping pills or other medications that affect sleep. All participants provided informed consent. A total of 20,184 online questionnaires were collected, of which 17,085 were valid, resulting in a response rate of 85%.

### 2.2 Instruments

An on-site survey was conducted using the Pittsburgh Sleep Quality Index Scale (PSQI), the Mobile Phone Involvement Questionnaire (MPIQ), and a basic information form. Two options were provided for completing the questionnaire: one using the most popular survey software in China^1^ and the other using a paper questionnaire. In both cases, participants were required to immediately show the completed questionnaire to the test personnel on-site. Additionally, although the questionnaires were filled out anonymously, we collected participants’ student IDs in the basic information section to exclude duplicate responses. Height and weight were measured on-site to calculate BMI. Preliminary survey results indicated a Cronbach’s alpha coefficient of 0.834, demonstrating good internal consistency of the questionnaire. Additionally, the analysis of the variance of common factors showed a cumulative variance contribution rate of 63.23%, further confirming the validity of the questionnaire structure.

#### 2.2.1 BMI

This study investigated the participants’ basic information including gender, age, height, and weight. The BMI was calculated using the internationally common formula: BMI = weight (kg)/height^2^ (m^2^) (41). According to the BMI classification standards developed for the Asian population (42), participants were categorized into ordinary (18.5 ≤ BMI ≤ 22.9 kg/m^2^), overweight (23 ≤ BMI ≤ 24.9 kg/m^2^), and obese (BMI ≥ 25 kg/m^2^) groups.

#### 2.2.2 Mobile phone involvement questionnaire

The Chinese version of the Mobile Phone Involvement Questionnaire, adapted by Lin et al. (43), was used to evaluate the degree of mobile phone involvement among university students. The scale consists of 8 items, scored on a 7-point scale (from 1: completely disagree to 7: completely agree). The total score ranges from 8 to 56, with a total score < 32 indicating no mobile phone involvement and ≥ 32 indicating mobile phone involvement. The Cronbach’s alpha coefficient for this study was 0.84.

#### 2.2.3 Sleep quality

The Pittsburgh Sleep Quality Index (PSQI) Scale, revised by Liu et al. (44), was used to assess the sleep quality of the participants. The scale comprises 7 dimensions (subjective sleep quality, sleep latency, sleep duration, sleep efficiency, sleep disturbances, use of sleeping medication, and daytime dysfunction), with a total of 19 items. The total score ranges from 0 to 21, with higher scores indicating poorer sleep quality. A PSQI total score > 7 was used to determine poor sleep quality. The Cronbach’s alpha coefficient for this study was 0.83, indicating good reliability and validity of the questionnaire.

### 2.3 Quality control

The initial draft of the questionnaire underwent revisions by three psychology experts and two health counselors. Additionally, feedback was gathered from a random sample of 50 university students during a pilot survey to enhance the questionnaire content. Through this iterative process, a high-quality survey tailored to the needs of this study was developed. Prior to formal administration, uniform training was provided to the surveyors. Data collection was conducted by two proficient research postgraduates who entered and cross-checked the data. Invalid responses were filtered out, and a dual-entry method was employed to establish the database, ensuring the accuracy and credibility of the data.

### 2.4 Statistical analysis

The database was established using EpiData 3.1, and data analysis was performed using SPSS 26.0 and GraphPad Prism 8.0. Descriptive statistics methods were used to summarize the basic information, including the calculation of means ( $\bar{x}$ ), standard deviation (SD), numbers and percentage of participants. A Pearson correlation analysis was employed to primarily investigate the relationship among mobile phone involvement, BMI, and sleep quality. A two-way ANOVA was employed to analysis the effect of BMI levels and mobile phone involvement on sleep quality and their interactive effect. A multiple linear regression was employed to identify the predictive effects of BMI levels and mobile phone involvement on sleep quality. In the regression analysis, all categorical variables were pre-processed using dummy variable encoding. Additionally, age and gender were included as covariates in the regression model.

## 3 Results

### 3.1 Descriptive statistics

The basic characteristics were showed in Table 1, in which the sex, age, BMI level, mobile phone involvement, and sleep quality of participants was included. Among the total of 17,085 individuals, 51.04% were male (8721) and 48.96% were female (8364). In terms of age groups, minors comprised 5.81% (993), while adults accounted for 94.19% (18 years old: 25.56%, 19 years old: 29.29%, 20 years old: 26.12%, 21 years old: 13.22%). Regarding BMI groups, 65.68% were classified as normal weight (11222), 15.87% as overweight (2712), and 18.45% as obese (3151). Mobile phone involvement was reported by 35.87% of participants (6128), while 64.13% (10957) reported no involvement. Finally, sleep quality assessment indicated that 42.06% had good sleep quality (7186), whereas 57.94% reported poor sleep quality (9899).

**Table 1:** Basic characteristics of the participants.

Variables	Parameters
Sex, *N* (%)	
Male	8,721 (51.04%)
Female	8,364 (48.96%)
Age, years old ( $\bar{x}\pm SD$ )	18.8 ± 1.02
Age groups, *N* (%)	
<18 years old	993 (5.81%)
18 years old	4,367 (25.56%)
19 years old	5,005 (29.29%)
20 years old	4,462 (26.12%)
21 years old	2,258 (13.22%)
BMI groups, *N* (%)	
Normal weight	11,222 (65.68%)
Overweight	2,712 (15.87%)
Obese	3,151 (18.45%)
Mobile phone involvement, *N* (%)	
No involvement	10,957 (64.13%)
Involvement	6,128 (35.87%)
Sleep quality, *N* (%)	
Good sleep quality	7,186 (42.06%)
Poor sleep quality	9,899 (57.94%)

### 3.2 The correlation between BMI levels, mobile phone involvement, and sleep quality

The result of correlation analysis (Table 2) showed that the scores of PSQI had significant positive correlations with both their mobile phone involvement (*R* = 0.22, *p* < 0.01) and BMI groups (*R* = 0.33, *p* < 0.01), which indicated that sleep quality are negatively correlated with mobile phone involvement and BMI.

**Table 2:** Correlation analysis.

Items		Mobile phone involvement	BMI groups	PSQI
Mobile phone involvement	*R*	1	0.08^**^	0.22^**^
	*p*		0.00	0.00
BMI levels	*R*		1	0.33^**^
	*p*			0.00
PSQI	*R*			1
	*p*			

^**^p < 0.01.

### 3.3 The impact of mobile phone involvement and BMI on sleep quality

Table 2 showed the results of two-way ANOVA conducted on the sleep quality. Significant effects were observed in both mobile phone involvement (*F* = 451.87^***^, *η*^2^ = 0.026) and BMI levels (*F* = 1527.01^***^, *η*^2^ = 0.15), while on interaction was observed between them.

**Figure 1:**
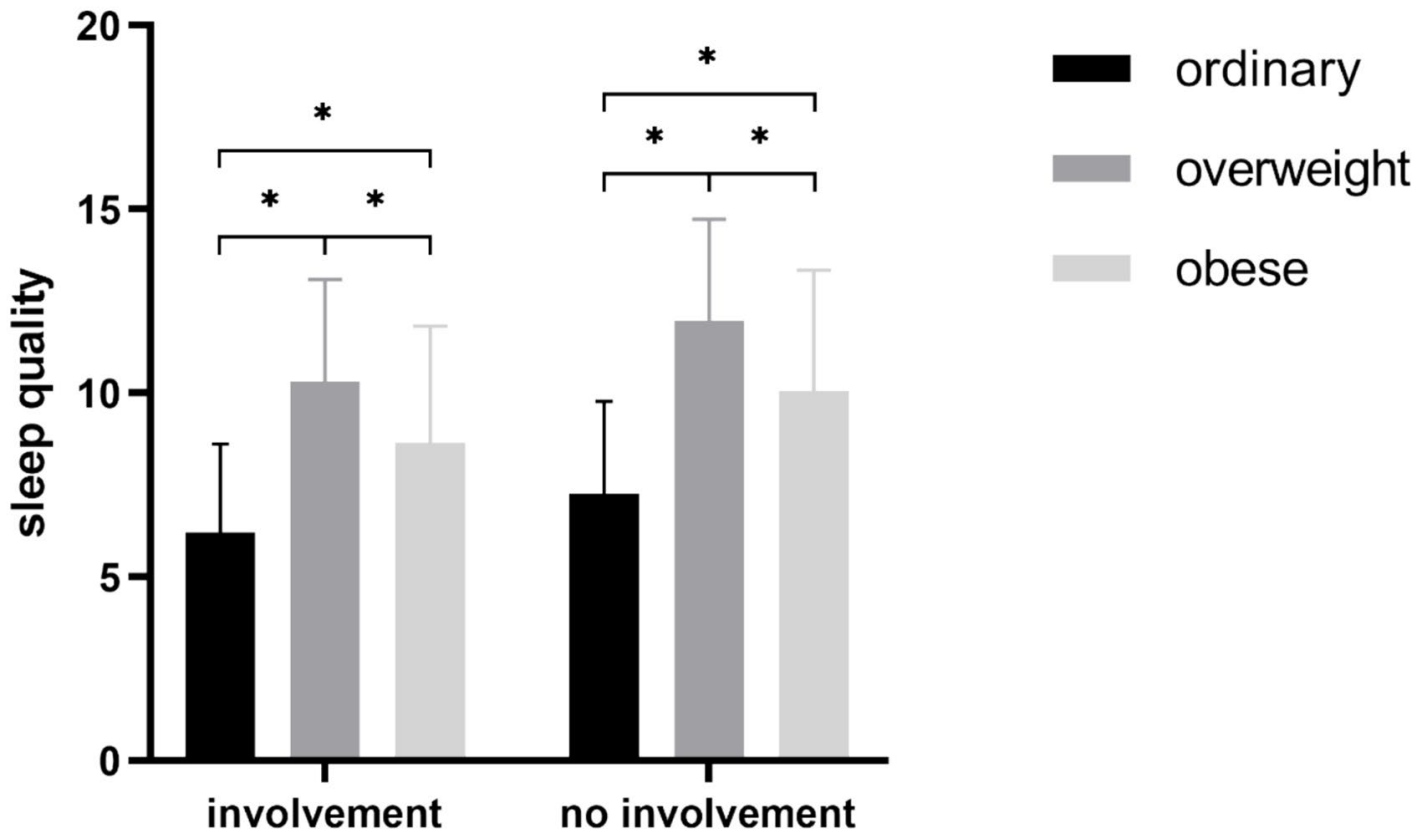
Comparisons of different BMI levels. ^*^p < 0.05, ^**^p < 0.01, ^***^p < 0.001.

Given the significant effect of BMI on sleep quality, a further comparison of the sleep quality among the three BMI groups was conducted. The results (Figure 1) showed that the sleep quality of ordinary group is significantly better (*p* < 0.05) than that of both overweight group and obese group, and sleep quality of overweight group is the significantly poorer than that of the other two groups (*p* < 0.05).

### 3.4 Linear regression of sleep quality based on BMI levels and mobile involvement

After included age groups and sex as the covariates, the results of the regression analysis (Table 3) indicated that BMI and mobile phone involvement can significantly predict sleep quality (*F* = 801.85, *p* < 0.001, *R*^2^ = 0.19). The intercept was 10.213 (*t* = 89.71, *p* < 0.001). Both BMI groups (*β =* −2.69, *t* = −52.90, *p* < 0.001) and Mobile phone involvement (*β =* −1.34, *t* = −28.25, *p* < 0.001) had a negative coefficient, suggesting that increased involvement and BMI is significantly associated with poorer sleep quality.

**Table 3:** The effect of mobile phone involvement and BMI on sleep quality.

Mobile phone involvement	BMI groups	Sleep quality
No involvement	Ordinary	6.21 ± 2.40
	Overweight	10.30 ± 2.79
	Obese	8.64 ± 3.184
Involvement	Ordinary	7.26 ± 2.52
	Overweight	11.95 ± 2.78
	Obese	10.06 ± 3.28
Effect of mobile phone involvement	*F*	451.87^***^
	*η^2^*	0.026
Effect of BMI levels	*F*	1527.01^***^
	*η* ^2^	0.15
Mobile phone involvement*BMI group	*F*	2.51
	*η* ^2^	0.00

^***^p < 0.001.

## 4 Discussion

In this study, we conducted a large-scale cross-sectional survey involving 17,085 participants to comprehensively examine the BMI, mobile phone involvement, and sleep quality among Chinese university students. Furthermore, our study included participants from major regions of eastern China, central China, and western China. To our knowledge, this may be one of the most comprehensive investigations among Chinese university students (Table 4).

**Table 4:** Regression model parameter.

Factor		*β*	*t*	*p*	VIF
Independent variable	Intercept	10.14	89.71^***^	0.00	
	Mobile phone involvement	−1.34	−28.25^***^	0.00	1.007
	BMI groups	−2.69	−52.90^***^	0.00	1.064
COVARIATE	<18 years old	0.34	6.99^***^	0.00	1.114
	≥18 years old	0.05	0.52	0.60	1.082
	Male	−0.25	−5.27^***^	0.00	1.038
	Female	0.00			
DW test	1.84				
*R* ^2^	0.19				
*F*	801.85^***^				
*p*	0.00				

^***^p < 0.001.

As reported by the WHO, the global overall prevalence of obesity has reached 12.5%, with 160 million of those being young people (45). The report on the physical fitness monitoring of the Chinese population shows that the overweight/obesity rate among females aged 20 to 25 can reach 20 to 30%, while for males, it can reach 40–50% (46). However, there are currently few studies, especially large-scale cross-sectional surveys, reporting on the overweight and obesity rates among Chinese university students. In response, our study found that the overweight rate among university students in China was 15.87%, while the obesity rate was 18.45%. The higher obesity rate indicates that the university student in China have become a high-risk group for obesity, warranting increased attention. Furthermore, our results demonstrated that 35.87% of the participants exhibited mobile phone involvement, which was inconsistent with the result of another cross-sectional survey (52.8%, with 2,741 university students from one school) (47). The differences in sleep quality assessment tools and the specificity of a single-school sample may explain this variation. Overall, these results indicated that Chinese university students exhibited a high level of mobile phone involvement, which is significantly higher than that in other Asian countries, such as Japan (12%) (48) and South Korea (19.9%) (49). These findings suggest that Chinese university students may be more prone to experiencing mobile phone involvement. To date, only one research has revealed the bidirectional relationship between loneliness, depressive symptoms, and mobile phone involvement among Chinese university students (50). However, this evidence remains insufficient to explained these cross-nation differences. Additionally, it is noteworthy that there is currently limited research on the physiological factors underlying smartphone involvement among Chinese university students, which also merits further investigation.

As for sleep quality, our results showed an extremely high prevalence of poor sleep quality (57.64%) among Chinese university students, which was significantly higher than the reports by existing studies [19% by Lu et al. (51) and 18.5% by Jiang et al. (52)]. A few potential factors may account for this heterogeneity, first, the significant difference in sample size may lead to variations in these results. Second, students from different schools may experience varying academic and social pressures, making the choice of school an important factor. Furthermore, existing studies mostly focus on a specific year of college students rather than multiple years. The differences in academic environments related to grades levels may also be an important reason for this heterogeneity. However, this findings on sleep quality remains alarming, despite evidence showing a downward trend in the sleep quality of Chinese university students over the past 20 years (51, 53).

Based on these findings, we investigated the relationship among the BMI groups, mobile phone involvement, and sleep quality. The result of correlation analysis indicated that both BMI and mobile phone involvement have significant negative correlation with sleep quality. However, these results can not imply causation, suggesting the need for further research to explore whether BMI and mobile phone involvement may contribute to reduced sleep quality. Furthermore, we also observed a significant positive correlation between BMI groups and mobile phone involvement, indicating that there may be an interaction effect of these two factors on the sleep quality. Therefore, we further examine the effect of BMI groups and mobile phone involvement on sleep quality. The results of ANOVA partially support the hypothesis that both BMI groups and mobile phone involvement showed extremely significant effect (*p* < 0.001) on sleep quality. To our surprise, no significant interaction was observed between them, suggesting that these two factors may be independently correlated with sleep quality among Chinese university students. Interestingly, we also found that sleep quality and BMI levels may not follow a straightforward negative relationship, as our participants showed that overweight individuals had significantly poorer sleep quality than obese individuals. So far, existed evidence has demonstrated that diet-driven weight gain is an important contributor to reduced sleep quality (54–56), which may be inconsistent with our results. However, Eid et al. found that the obesity category (normal-weight/ overweight/obesity) cannot predict any aspect of sleep quality (57), which may support the our results. Furthermore, a recent national survey conducted on the Chinese older adults showed that overweight/obese individuals may have a better sleep quality compared to older adults with normal weight (58). In summary, current evidence has indicated that the casual relationship between sleep quality and BMI remains unclear. Further exploration of this relationship in future may be beneficial for improving both weight management and sleep quality among university students.

Due to these independent effects, we conducted a linear regression to evaluate the explanatory power of the two on sleep quality. As reported by multiple researches, the difference of gender and the resulting lifestyle preference are closely related to sleep quality among students (59–61). Similarly, age is also significantly related to sleep, particularly short sleep patterns of university students (62). Therefore, we included gender and age as covariates in the regression analysis to investigate the correlation among BMI, smartphone involvement, and sleep quality more precisely. In our regression model, the low VIFs indicates that our investigations based on a large sample survey are relatively robust. The results indicate that BMI and mobile phone dependency have a significant predictive effect on sleep quality (*p* < 0.001), accounting for 19% of the variance. It is well known that sleep is influenced by various factors such as diet, mood, medication, and lifestyle (63). However, there is currently limited quantitative evidence to support this. In this regard, the predictive model provided by our study may help reveal part of the relative weight of these factors. Besides, in our findings, BMI level exhibited a stronger correlation with sleep quality compared to mobile phone effect (*β*_BMI_ vs. β _mobile phone involvement_: –52.90 vs. -28.25).

For BMI, studies have showed that an increase of 6 units BMI can resulted in four time greater risk of OSA (64, 65), specifically, The disruption of pro-inflammatory factors (e.g., IL-6, IL-12, TNF-*α*) caused by excessive visceral fat has been identified as a significant risk factor for sleep disorders (66, 67). Among the obese individuals, unhealthy dietary habits, such as high intake of carbohydrates and saturated fatty acids, have been shown to impair sleep (68). In contrast, the intake of polyunsaturated fatty acids has been shown to promote melatonin production and facilitate normal sleep (69). Notably, although our study mainly investigated the effect of BMI on sleep quality, the poor sleep is also proved to be a predisposing risk factor of obesity. As reported by a four-year follow-up prospective study conducted on 14,000 young adults, short sleep persistent exposure leads to an 1.45 times increase in obesity and elevated WC development (70). Regarding mobile phone involvement, our results are in consistent with the current evidence. Brautsch et al. revealed a closely correlation between mobile phone use with later bedtime and daytime tiredness by integrated 42 relevant studies (71), and our study provides evidence from Chinese university students that further this finding. However, we found limited evidence about the impact of poor sleep on the mobile phone usage, which may help understanding the complex relationship between sleep behavior and mobile phone involvement.

## 5 Limitations

The main limitation of this study is that the cross-sectional design makes it difficult to establish the causal relationship between BMI, mobile phone dependency, and sleep quality among Chinese university students. Although we found limited evidence, we must acknowledge the possibility of indirect reverse causation. For instance, Hartanto et al. summarize the current clues regarding the influence of social media on depression, highlighting the reverse effect of depression on social media use (72). Furthermore, depression has been shown to be highly correlated with sleep disorders (73), making it important to consider the impact of poor sleep quality on smartphone use as well. Additionally, students may also use their phones to cope with difficulties in falling asleep. Therefore, we recommend conducting longitudinal studies in the future to further confirm the directional relationships among the three variables. Besides, this study utilized self-reported questionnaire tools to assess mobile phone usage and sleep quality, which are prone to social desirability and recall biases. Especially regarding mobile phone involvement, the accuracy of self-reports may be weaker due to the extensive use of digital media usage (74). Consequently, we recommend that future research further categorize participants and utilize tools such as accelerometers for detailed validation of the related issues.

Due to the challenges of conducting field surveys within a limited timeframe, we must acknowledge that the control variables included in our model are limited. While the large sample size can help balance some fundamental systematic errors, certain potential factors may also affect the results. Specifically, mental health status factors such as individual anxiety and depression, have been proven to be risk factors for sleep quality (69, 75, 76). In contrast, physical activity has been shown to be a protective factor (77). Besides, the varying academic pressure faced by students in different grades (78), as well as their differing socioeconomic status (79) may also be critical confounders that we have not considered.

## 6 Conclusion

The main findings of this study indicate that higher BMI levels and mobile phone involvement are independently correlated with sleep quality among Chinese university students. Furthermore, the study highlights a significant prevalence of overweight and obesity, alongside high rates of mobile phone dependency, with more than half of the participants reporting poor sleep quality. These results underscore the need for targeted interventions addressing both weight management and mobile phone usage to improve sleep health in this population.

## Data Availability

The raw data supporting the conclusions of this article will be made available by the authors, without undue reservation.

## Footnotes

^1^ http://www.wjx.cn/
